# Vaccination with long NY-ESO-1 79-108 peptide and CpG-B leads to robust activation of CD4 and CD8 T cell responses in stage III/IV melanoma patients, and a new HLA-DR7 epitope

**DOI:** 10.1186/2051-1426-3-S2-P437

**Published:** 2015-11-04

**Authors:** Petra Baumgartner, Carla Costa Nunes, Amélie Cachot, Hélène Maby-El Hajjami, Laurène Cagnon, Marion Braun, Laurent Derré, Jean-Paul Rivals, Donata Rimoldi, Emanuela Romano, Olivier Michielin, Pedro Romero, Camilla Jandus, Daniel E Speiser

**Affiliations:** 1 Ludwig Center for Cancer Research at the University of Lausanne and Department of Oncology, University Hospital of Lausanne, Lausanne, Switzerland; 2 Ludwig Cancer Research Center, University of Lausanne, Lausanne, Switzerland; 3 Department of Oncology, Ludwig Cancer Research Center, University of Lausanne, Lausanne, Switzerland; 4 Department of Oncology, University Hospital Center (CHUV), Lausanne, Switzerland; 5 Miltenyi Biotech GmbH, Bergisch Galdbach, Germany; 6 Urology Research Unit, Urology Department, University Hospital Center (CHUV), Lausanne, Switzerland; 7 Department of Otolaryngology, Head and Neck Surgery, University Hospital Center (CHUV), Lausanne, Switzerland; 8 Centre Hospitalier Universitaire Vaudois, Lausanne, Switzerland

## 

Although promising, the combination of long synthetic peptides and CpG-B oligodeoxynucleotides has not yet been tested as cancer vaccine. In this Phase I trial, 19 patients received a mean of 8 (range 1-12) monthly vaccines s.c. composed of the long synthetic NY-ESO79-108 peptide and CpG-B (PF-3512676), emulsified in Montanide ISA-51. In 18/18 evaluable patients, vaccination induced responses of both CD8 and CD4 T cells, starting early after initiation of immunotherapy and lasting for many months. The T cells responded antigen-specifically, with strong secretion of IFNγ and TNFα, irrespective of patient's HLAs. The most immunogenic region of the vaccine peptide was the NY-ESO-183-97 sequence, inducing HLA-DR or -DP restricted CD4 T cell responses in all patients tested. We discovered a novel and highly immunogenic epitope (HLA-DR7/NY-ESO-187-99); 5/5 HLA-DR7+ patients generated strong CD4 T cell responses, as detected directly *ex-vivo* with fluorescent multimers. Thus, vaccination with the long synthetic NY-ESO-179-108 peptide combined with the strong immune adjuvant CpG-B, a TLR-9 agonist, induced integrated, robust and functional CD8 and CD4 T cell responses in melanoma patients, supporting the further development of this immunotherapeutic approach.

